# Clinical Practice Preferences for Glaucoma Surgery in Japan in 2024

**DOI:** 10.3390/jcm14062039

**Published:** 2025-03-17

**Authors:** Kentaro Iwasaki, Shogo Arimura, Yoshihiro Takamura, Masaru Inatani

**Affiliations:** Department of Ophthalmology, Faculty of Medical Sciences, University of Fukui, Fukui 910-1193, Japan; kenkentaro0329@yahoo.co.jp (K.I.); leosunshine33@gmail.com (S.A.); ytakamura@hotmail.com (Y.T.)

**Keywords:** glaucoma surgery, surgical preferences, trabeculectomy, PreserFlo MicroShunt, long-tube shunt surgery, minimally invasive glaucoma surgery

## Abstract

**Objectives:** This study evaluated the clinical preferences of glaucoma specialists regarding glaucoma surgery and postoperative management in Japan in 2024. **Methods:** A survey about clinical practice preferences regarding glaucoma surgery and postoperative care was administered among 50 glaucoma specialists who were councilors in the Japan Glaucoma Society. **Results:** Minimally invasive glaucoma surgery (MIGS) plus phacoemulsification was the most preferred procedure for nonoperated mild to moderate cases of primary open-angle glaucoma (POAG) (94.6%) and normal tension glaucoma (NTG) (67.3%) associated with cataract. Microhook surgery was the most preferred among the MIGS procedures. Meanwhile, PreserFlo MicroShunt (PMS) surgery is emerging as a popular option for cases of POAG and NTG, especially in advanced-stage pseudophakic eyes that underwent prior corneal incision phacoemulsification (40.1%). Long-tube shunt surgeries were predominantly preferred for POAG after two failed trabeculectomies (69.4%) and for neovascular glaucoma with prior vitrectomy after a failed trabeculectomy (73.0%). Among long-tube shunt surgeries, the Ahmed glaucoma valve (AGV) was preferred over the Baerveldt glaucoma implant. Trabeculectomy required the most frequent follow-up visits within the first postoperative year, whereas PMS and long-tube shunt surgeries required comparatively fewer follow-up visits. Overall, MIGS involved less frequent follow-up visits versus filtering surgeries. **Conclusions:** MIGS is currently the procedure of choice for primary glaucoma surgery in Japan. Among glaucoma specialists of the Japan Glaucoma Society, PMS surgery is becoming popular for cases of POAG and NTG. Refractory glaucoma is commonly treated with long-tube shunt surgeries, especially the AGV.

## 1. Introduction

Trabeculectomy is a filtering surgery widely performed worldwide for cases of glaucoma with medically uncontrollable intraocular pressure (IOP), but is commonly associated with severe postoperative complications related to visual impairment [1]. Trabeculectomy is highly effective in lowering IOP; however, due to its high complication rate and complex postoperative management, it is a procedure that is preferably avoided when possible. To address this, less invasive surgical techniques have been recently developed, with a variety of procedures now available. For example, the iStent inject W (Glaukos Corporation, San Clemente, CA, USA) and the Hydrus Microstent (HMS) (Alcon Inc., Geneva, Switzerland) were newly approved for use in Japan. The iStent inject W was first approved in combination with phacoemulsification in October 2019, and then as a standalone procedure in July 2024. Similarly, the HMS plus phacoemulsification was approved in June 2024. Therefore, minimally invasive glaucoma surgery (MIGS), which includes these procedures, has gained global popularity as a primary option for glaucoma surgery [2,3,4,5,6] due to its favorable safety profile and shorter recovery time [7,8]. Meanwhile, the PreserFlo MicroShunt (PMS) (Santen Pharmaceutical Company, Osaka, Japan) is a microinvasive filtration surgical device that was approved for use in Japan in February 2022. PMS surgery is a type of glaucoma filtration surgery that is less invasive than trabeculectomy because it does not require the creation of a scleral flap or iridectomy [9]. Additionally, long-tube shunt surgeries using a Baerveldt glaucoma implant (BGI) (Johnson & Johnson Vision, Jacksonville, FL, USA) and an Ahmed glaucoma valve (AGV) (New World Medical, Rancho Cucamonga, CA, USA) have also become increasingly popular for refractory glaucoma [4,10].

Several studies have surveyed surgical preferences for glaucoma in the United States and the United Kingdom [11,12,13], but no recent reports are available. In Japan, surgical preferences for glaucoma have not been recently evaluated. In 2019, we previously reported the clinical preferences of Japan Glaucoma Society specialists regarding glaucoma surgery and postoperative management, but this was not updated with recent advancements in the field [4]. Although recent studies have shown trends in surgical procedures for glaucoma in Japan using a nationwide administrative database [14,15], these do not capture the current specific clinical preferences of glaucoma specialists. Considering the rapid advancements in glaucoma surgery over the past five years, especially with the emergence of new surgical options, this study aimed to evaluate the updated clinical preferences of glaucoma specialists regarding the surgical and postoperative management of glaucoma in Japan in 2024. Given the lack of recent reports from the United States and the United Kingdom, this report provides a particularly valuable reference for surgical decision-making and the postoperative management of glaucoma in similar clinical scenarios.

## 2. Materials and Methods

Institutional review board approval was waived by the ethics committee of the University of Fukui Hospital, Fukui, Japan, because patients were not involved. Survey questions were emailed to 50 glaucoma specialists who were council members of the Japan Glaucoma Society in November 2024. Participants were asked about their years of experience as ophthalmologists and whether they were actively performing glaucoma surgeries at the time of the survey. Active surgeons were then presented with 20 clinical scenarios and asked to indicate their preferred surgical approach for each (Supplemental Table S1). Among these questions, the severity of glaucoma was classified based on the mean deviation (MD) score: mild to moderate (MD > −12 dB) and advanced (MD ≤ −12 dB). Each answer was assigned 1 point; if a participant selected multiple answers to a question, this 1 point was divided by the number of answers. Finally, participants were asked about the frequency of follow-up visits for each type of glaucoma surgery during the first postoperative year.

## 3. Results

The survey was completed by all 50 glaucoma specialists, with a mean of 30.5 ± 5.8 years (range: 14–42) of experience as ophthalmologists. Among them, 42 were actively performing glaucoma surgery at the time of the survey; these specialists answered questions regarding their preferences for glaucoma surgery. Supplemental Table S2 summarizes the overall surgical preferences for each type of glaucoma across all clinical settings.

### 3.1. Surgical Choices for Primary Open-Angle Glaucoma

Table 1 shows participants’ surgical choices for primary open-angle glaucoma (POAG) across eight clinical settings.

#### 3.1.1. Mild to Moderate Cases (MD > −12 dB)

For unoperated eyes with cataract, the most preferred procedure was microhook plus phacoemulsification (51.8%), followed by the iStent inject W plus phacoemulsification (18.1%). Overall, 94.6% preferred MIGS plus phacoemulsification as the first-line surgical approach for mild to moderate cases of POAG. For unoperated eyes without cataract and pseudophakic eyes, microhook surgery alone was the most commonly chosen procedure (50.8% and 48.4%, respectively) followed by PMS surgery alone (17.5% and 23.4%, respectively).

#### 3.1.2. Advanced Cases (MD ≤ −12 dB)

For unoperated eyes with cataract, the most preferred procedure was MIGS plus phacoemulsification (36.7%), but PMS surgery plus phacoemulsification was frequently selected as well (28.8%). For unoperated eyes without cataract, trabeculectomy alone was the predominant choice (52.0%). For pseudophakic eyes, the most commonly selected procedure was trabeculectomy (49.6%), followed by PMS surgery (40.1%). For pseudophakic eyes with a previous failed trabeculectomy, trabeculectomy remained the most frequently selected option (57.2%), but after two failed trabeculectomies, long-tube shunt surgeries were chosen, with a preference for the AGV (49.0%) followed by the BGI (20.4%).

### 3.2. Surgical Choices for Normal Tension Glaucoma

Table 2 summarizes participants’ surgical preferences for normal tension glaucoma (NTG) across six clinical settings.

#### 3.2.1. Mild to Moderate Cases (MD > −12 dB)

For unoperated eyes with cataract, MIGS plus phacoemulsification was preferred by 67.3%. Among the different types of MIGS, the most preferred was microhook surgery plus phacoemulsification (37.5%), followed by PMS surgery plus phacoemulsification (20.2%), while trabeculectomy plus phacoemulsification was less frequently preferred (7.1%).

#### 3.2.2. Advanced Cases (MD ≤ −12 dB)

For unoperated eyes with cataract, trabeculectomy plus phacoemulsification was the most preferred procedure (36.3%), followed by microhook surgery plus phacoemulsification (21.6%). In this scenario, 31.1% generally preferred MIGS plus phacoemulsification. For unoperated eyes without cataract, the most dominant choice was trabeculectomy alone (72.4%) followed by PMS surgery alone (19.1%). For pseudophakic eyes, the preferred procedure was trabeculectomy (69.4%) followed by PMS surgery (25.8%). For pseudophakic eyes with a previous failed trabeculectomy, trabeculectomy remained the most commonly selected procedure (75.0%), but after two failed trabeculectomies, long-tube shunt surgeries were chosen, with a preference for the AGV (34.1%) followed by the BGI (20.6%).

### 3.3. Surgical Choices for Uveitic Glaucoma

Table 3 outlines participants’ surgical choices for uveitic glaucoma (UG) across four clinical settings. For unoperated eyes with cataract, surgeons preferred trabeculectomy plus phacoemulsification (32.5%) followed by microhook surgery plus phacoemulsification (20.2%), and then trabeculectomy alone (20.2%). In such cases, 33.7% preferred MIGS plus phacoemulsification. For unoperated eyes without cataract, the dominant choice was trabeculectomy alone (62.7%), while microhook surgery and PMS surgery alone were preferred by 14.3% and 10.7%, respectively. For pseudophakic eyes, the preferred procedure was trabeculectomy (57.2%) followed by microhook surgery (13.9%) and PMS surgery (11.5%). For pseudophakic eyes with a previous failed trabeculectomy, trabeculectomy remained the dominant choice (53.7%) followed by AGV surgery (20.8%) and PMS surgery (13.1%).

### 3.4. Surgical Choices for Neovascular Glaucoma

Table 4 presents the surgical preferences of participants for neovascular glaucoma (NVG) in two clinical settings. For pseudophakic eyes with prior vitrectomy, the most frequently selected procedure was trabeculectomy (47.2%) followed by an AGV (34.1%). Conversely, for pseudophakic eyes with prior vitrectomy and a failed trabeculectomy, the preferred option was long-tube shunt surgery with an AGV (62.7%), while the preference for trabeculectomy decreased to 19.8%.

### 3.5. Postoperative Management

**Figure 1:**
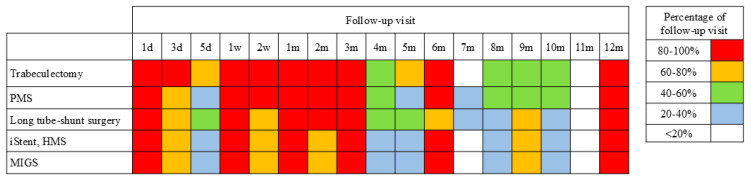
Frequency of postoperative follow-up for each type of glaucoma surgery. d, day; w, week; m, month; HMS, Hydrus Microstent; MIGS, minimally invasive glaucoma surgery; PMS, PreserFlo MicroShunt.

Figure 1 illustrates the follow-up intervals for each type of glaucoma surgery. After trabeculectomy, more than 80% of surgeons held follow-up sessions at 1 day, 3 days, 1 week, 2 weeks, 1 month, 2 months, and 3 months postoperatively, then every 2 or 3 months. After PMS surgery and long-tube shunt surgeries, more than 80% of surgeons held follow-up sessions at 1 day, 1 week, 2 weeks, 1 month, 2 months, and 3 months postoperatively, then every 2 or 3 months. These procedures tended to require fewer early postoperative visits compared to trabeculectomy. After the iStent, Hydrus, and other types of MIGS, more than 80% of surgeons held follow-up sessions at 1 day, 1 week, 1 month, 3 months, 6 months, and 1 year postoperatively. These findings suggest that fewer postoperative visits are required after MIGS compared to filtering surgery.

## 4. Discussion

This article provides a comprehensive overview of the current surgical preferences and postoperative management strategies of glaucoma specialists in Japan. The findings indicate a significant shift toward MIGS as the preferred primary surgical intervention, particularly in mild to moderate cases of POAG and NTG associated with cataracts. MIGS, when combined with cataract surgery, provides excellent IOP-lowering effects [16,17]. Therefore, it is a preferred option for patients with both glaucoma and cataracts. Among the different types of MIGS, microhook surgery was the most preferred option, similar to our previous survey’s findings [4]. This preference for microhook surgery could be attributed to its lower cost due to the reusable device, as well as its well-established effectiveness in clinical practice [18], making it an appealing option for many surgeons.

PMS surgery has also increased in popularity, particularly for advanced cases of POAG and NTG in pseudophakic eyes. Similar to long-tube shunt surgeries, PMS surgery causes greater endothelial cell loss when the tube is positioned closer to the corneal endothelium [19,20]. Therefore, it is likely preferred in pseudophakic eyes with a deeper anterior chamber rather than in phakic eyes. PMS surgery is frequently selected in cases wherein trabeculectomy was previously favored, indicating a trend toward the adoption of less invasive filtration surgery with potentially fewer complications [9,21]. Moreover, the preference for PMS surgery in advanced glaucoma cases without prior surgical intervention further underscores its perceived efficacy and safety among glaucoma specialists. However, because of its superior IOP-lowering effect versus PMS surgery [21], trabeculectomy remains the procedure of choice in cases requiring intense IOP reduction, particularly in advanced NTG eyes. In Japan, NTG is the most common type of glaucoma [22] wherein the effectiveness of trabeculectomy has been reported [23,24]. Furthermore, trabeculectomy remains the preferred surgical option for POAG and NTG eyes with a previously failed trabeculectomy, as well as for UG or NVG eyes as a primary glaucoma surgery. Thus, even with the shift toward less invasive surgical techniques, trabeculectomy continues to play a crucial role.

For refractory glaucoma cases, including those with multiple failed trabeculectomies, long-tube shunt surgeries remain the preferred choice, with the AGV being favored over the BGI. The results also suggest that long-tube shunt surgery with an AGV is preferred for NVG with prior vitrectomy and a previous failed trabeculectomy, thus reinforcing its role in managing complex and high-risk glaucoma cases. In contrast, our 2019 survey revealed that the BGI and AGV had comparable selection rates in similar scenarios [4]. This shift can be attributed to the safety and ease of the procedure with an AGV, despite having a weaker IOP-lowering effect than the BGI [25,26]. The AGV features a valved mechanism that provides immediate IOP control and reduces the risk of postoperative hypotony [25]. The AGV tends to cause less postoperative visual loss than the BGI [26]. Additionally, the AGV has a small plate, is easy to implant, and requires only one quadrant, which allows for potential additional glaucoma surgeries in the future.

There were notable variations in the frequency of postoperative follow-up depending on the type of surgery performed. Trabeculectomy requires the most frequent early postoperative visits, which is appropriate because of the higher risk of complications (e.g., bleb leaks, hypotony, and infections) and the need for proactive and reactive bleb management to achieve better postoperative outcomes [27,28]. In contrast, PMS and long-tube shunt surgeries require fewer early follow-up visits, likely due to their more predictable postoperative course. Meanwhile, MIGS procedures, including the iStent inject W and the HMS, require the least frequent follow-up because of their minimally invasive nature and rapid recovery time.

The trends observed in this study are consistent with global shifts in glaucoma surgical practice [2,3,5,6,29]. Surveys in the United States and the United Kingdom have similarly reported an increasing adoption of MIGS, shifting away from traditional trabeculectomy as a first-line surgical intervention. In Japan, both patients and surgeons have come to prefer less invasive procedures such as MIGS and PMS due to their safety, consistent IOP-lowering effects, and simplified postoperative management. However, the preference for trabeculectomy in advanced glaucoma cases remains strong among Japanese specialists. This suggests that despite the emergence of newer techniques, traditional filtering surgery continues to be a mainstay for managing severe glaucoma. In the United States, endoscopic cyclophotocoagulation (ECP) and micropulse transscleral cyclophotocoagulation (MP-TSCPC) are types of MIGS that are commonly used as primary surgical treatments for glaucoma [2,6,30,31]. In Japan, ECP is currently available at a limited number of facilities and is primarily used for extremely refractory cases of glaucoma. Meanwhile, MP-TSCPC is also used for eyes with refractory glaucoma in Japan, but its surgical indication remains low.

Despite these valuable insights, this study had certain limitations. First, the study population included only 50 glaucoma specialists who are council members of the Japan Glaucoma Society, and thus these data may not fully represent the broader population of glaucoma surgeons in Japan. Second, because the survey was conducted shortly after the approval of new procedures, namely the standalone iStent inject W and the HMS, some of these specialists may have limited experience with them. Third, this study relied on survey responses rather than clinical outcome data. There may have been bias regarding individual preferences, as objective efficacy metrics were not considered. In actual clinical practice, the choice of surgery varies based on patient-specific factors such as age, daily activities, socioeconomic status, access to care, and home environment. Fourth, we did not inquire about the reasons for selecting specific devices within similar surgical procedures (e.g., among MIGS or long-tube shunt surgeries), so the details of surgical preferences remain unclear. Future studies incorporating real-world patient characteristics, clinical outcomes, and long-term follow-up data can provide a more comprehensive understanding of the different types of glaucoma surgery.

In conclusion, this study highlights the evolving trends in glaucoma surgery among Japanese specialists. Notably, there was a strong preference for MIGS in mild to moderate cases, increasing adoption of PMS for advanced cases, and continued reliance on long-tube shunt surgeries for refractory cases. These findings provide valuable guidance for clinical decision-making and highlight the importance of continued research and innovation in the surgical management of glaucoma.

## Figures and Tables

**Table 1:** Surgical preferences for primary open-angle glaucoma.

Type of Glaucoma Surgery	Unoperated Eyes with Cataract		Unoperated Eyes Without Cataract		Pseudophakic Eyes with a Corneal Incision Phaco		Pseudophakic Eyes with a Previous Failed Trabeculectomy	Pseudophakic Eyes with Two Failed Trabeculectomies
	Mild to Moderate (%)	Advanced (%)	Mild to Moderate (%)	Advanced (%)	Mild to Moderate (%)	Advanced (%)	Advanced (%)	Advanced (%)
Microhook+Phaco	51.8	24.8	2.4	2.4	0.0	0.0	0.0	0.0
Microhook	0.0	0.0	50.8	5.9	48.4	4.7	0.8	0.8
iStent inject W+Phaco	18.1	3.3	1.2	1.2	0.0	0.0	0.0	0.0
iStent inject W	0.0	0.0	0.8	0.8	0.8	0.8	0.0	0.0
KDB+Phaco	7.1	3.6	0.0	0.0	0.0	0.0	0.0	0.0
KDB	0.0	0.0	9.5	0.0	7.2	0.0	0.0	0.0
Suture trabeculotomy+Phaco	6.7	1.4	0.0	0.0	0.0	0.0	0.0	0.0
Suture trabeculotomy	0.0	0.0	6.7	1.6	7.9	1.6	0.8	0.6
HMS+Phaco	6.1	2.4	0.0	0.0	0.0	0.0	0.0	0.0
Other MIGS+Phaco	4.8	1.2	0.0	0.0	0.0	0.0	0.0	0.0
Other MIGS	0.0	0.0	6.0	0.8	4.8	0.8	0.0	0.0
PMS+Phaco	4.8	28.8	0.0	0.0	0.0	0.0	0.0	0.0
PMS	0.0	1.6	17.5	32.9	23.4	40.1	18.2	7.1
Trabeculectomy+Phaco	0.0	20.8	0.0	0.0	0.0	0.0	0.0	0.0
Trabeculectomy	0.6	9.7	5.1	52.0	7.5	49.6	57.2	15.7
ExPRESS+Phaco	0.0	2.4	0.0	0.0	0.0	0.0	0.0	0.0
ExPRESS	0.0	0.0	0.0	2.4	0.0	2.4	2.4	0.0
AGV	0.0	0.0	0.0	0.0	0.0	0.0	10.7	49.0
BGI	0.0	0.0	0.0	0.0	0.0	0.0	4.8	20.4
Bleb revision or needling	0.0	0.0	0.0	0.0	0.0	0.0	5.1	1.6
MP-TSCPC	0.0	0.0	0.0	0.0	0.0	0.0	0.0	4.8

AGV, Ahmed glaucoma valve; BGI, Baerveldt glaucoma implant; HMS, Hydrus Microstent; KDB, Kahook dual blade; MIGS, minimally invasive glaucoma surgery; MP-TSCPC, micropulse transscleral cyclophotocoagulation; Phaco, phacoemulsification; PMS, PreserFlo MicroShunt.

**Table 2:** Surgical preferences for normal tension glaucoma.

Type of Glaucoma Surgery	Unoperated Eyes with Cataract		Unoperated Eyes Without Cataract	Pseudophakic Eyes with a Corneal Incision Phaco	Pseudophakic Eyes with a Previous Failed Trabeculectomy	Pseudophakic Eyes with Two Failed Trabeculectomies
	Mild to Moderate (%)	Advanced (%)	Advanced (%)	Advanced (%)	Advanced (%)	Advanced (%)
Microhook+Phaco	37.5	21.6	1.2	0.0	0.0	0.0
Microhook	0.0	0.0	4.5	0.8	0.8	0.8
iStent inject W+Phaco	14.5	4.1	1.2	0.0	0.0	0.0
iStent inject W	0.0	0.0	0.8	0.8	0.0	0.0
KDB+Phaco	4.8	2.4	0.0	0.0	0.0	0.0
Suture trabeculotomy+Phaco	2.0	0.6	0.0	0.0	0.0	0.0
Suture trabeculotomy	0.0	0.0	0.8	0.8	0.8	0.8
HMS+Phaco	6.1	2.4	0.0	0.0	0.0	0.0
Other MIGS+Phaco	2.4	0.0	0.0	0.0	0.0	0.0
Phaco only	2.4	0.0	0.0	0.0	0.0	0.0
PMS+Phaco	20.2	17.3	0.0	0.0	0.0	0.0
PMS	2.4	0.8	19.1	25.8	11.1	9.9
Trabeculectomy+Phaco	7.1	36.3	0.0	0.0	0.0	0.0
Trabeculectomy	0.6	12.1	72.4	69.4	75.0	18.7
ExPRESS+Phaco	0.0	2.4	0.0	0.0	0.0	0.0
ExPRESS	0.0	0.0	0.0	2.4	2.4	2.4
AGV	0.0	0.0	0.0	0.0	2.4	34.1
BGI	0.0	0.0	0.0	0.0	2.4	20.6
Bleb revision or needling	0.0	0.0	0.0	0.0	5.1	9.5
MP-TSCPC	0.0	0.0	0.0	0.0	0.0	3.2

AGV, Ahmed glaucoma valve; BGI, Baerveldt glaucoma implant; HMS, Hydrus Microstent; KDB, Kahook dual blade; MIGS, minimally invasive glaucoma surgery; MP-TSCPC, micropulse transscleral cyclophotocoagulation; Phaco, phacoemulsification; PMS, PreserFlo MicroShunt.

**Table 3:** Surgical preferences for uveitic glaucoma.

Type of Glaucoma Surgery	Unoperated Eyes with Cataract	Unoperated Eyes Without Cataract	Pseudophakic Eyes	Pseudophakic Eyes with a Previous Failed Trabeculectomy
Microhook+Phaco	20.2	1.2	0.0	0.0
Microhook	2.4	14.3	13.9	3.2
iStent inject W	0.0	0.0	0.8	0.0
KDB+Phaco	4.8	0.0	0.0	0.0
KDB	0.0	2.4	2.4	0.0
Suture trabeculotomy+Phaco	5.1	0.0	0.0	0.0
Suture trabeculotomy	0.0	4.3	5.1	0.6
HMS+Phaco	1.2	0.0	0.0	0.0
Other MIGS+Phaco	2.4	1.2	0.0	0.0
Other MIGS	0.0	2.4	2.4	0.0
PMS+Phaco	7.2	0.0	0.0	0.0
PMS	2.4	10.7	11.5	13.1
Trabeculectomy+Phaco	32.5	0.0	0.0	0.0
Trabeculectomy	20.2	62.7	57.2	53.7
AGV+Phaco	1.6	0.0	0.0	0.0
AGV	0.0	0.8	7.5	20.8
BGI	0.0	0.0	0.0	4.8
Bleb revision or needling	0.0	0.0	0.0	3.8

AGV, Ahmed glaucoma valve; BGI, Baerveldt glaucoma implant; HMS, Hydrus Microstent; KDB, Kahook dual blade; MIGS, minimally invasive glaucoma surgery; Phaco, phacoemulsification; PMS, PreserFlo MicroShunt.

**Table 4:** Surgical preferences for neovascular glaucoma.

Type of Glaucoma Surgery	Pseudophakic Eyes with Prior Vitrectomy and Panretinal Photocoagulation	Pseudophakic Eyes with Prior Vitrectomy, Panretinal Photocoagulation, and a Previous Failed Trabeculectomy
PMS	8.4	4.8
Trabeculectomy	47.2	19.8
ExPRESS	1.2	0.0
AGV	34.1	62.7
BGI	9.1	10.3
Bleb revision or needling	0.0	2.4

AGV, Ahmed glaucoma valve; BGI, Baerveldt glaucoma implant; PMS, PreserFlo MicroShunt.

## Data Availability

Data are fully available upon reasonable request to the corresponding author.

## Supplementary Materials

The following supporting information can be downloaded at: https://www.mdpi.com/article/10.3390/jcm14062039/s1, Table S1. Which glaucoma surgeries had you performed for the following clinical scenarios in 2024? Table S2: Surgical preferences for each type of glaucoma across all clinical settings.

## References

[1] Costa V.P., Smith M., Spaeth G.L., Gandham S., Markovitz B. (1993). Loss of visual acuity after trabeculectomy. Ophthalmology.

[2] Boland M.V., Corcoran K.J., Lee A.Y. (2021). Changes in performance of glaucoma surgeries 1994 through 2017 based on claims and payment data for United States Medicare beneficiaries. Ophthalmol. Glaucoma.

[3] Luebke J., Boehringer D., Anton A., Daniel M., Reinhard T., Lang S. (2021). Trends in surgical glaucoma treatment in Germany between 2006 and 2018. Clin. Epidemiol..

[4] Iwasaki K., Arimura S., Takamura Y., Inatani M. (2020). Clinical practice preferences for glaucoma surgery in Japan: A survey of Japan Glaucoma Society specialists. Jpn. J. Ophthalmol..

[5] Rathi S., Andrews C.A., Greenfield D.S., Stein J.D. (2021). Trends in Glaucoma Surgeries Performed by Glaucoma Subspecialists versus Nonsubspecialists on Medicare Beneficiaries from 2008 through 2016. Ophthalmology.

[6] Yang S.A., Mitchell W., Hall N., Elze T., Lorch A.C., Miller J.W., Zebardast N., IRIS^®^ Registry Data Analytics Consortium (2021). Trends and usage patterns of minimally invasive glaucoma surgery in the United States: IRIS^®^ registry analysis 2013–2018. Ophthalmol. Glaucoma.

[7] Yook E., Vinod K., Panarelli J.F. (2018). Complications of micro-invasive glaucoma surgery. Curr. Opin. Ophthalmol..

[8] Rosdahl J.A., Gupta D. (2020). Prospective studies of minimally invasive glaucoma surgeries: Systematic review and quality assessment. Clin. Ophthalmol..

[9] Batlle J.F., Fantes F., Riss I., Pinchuk L., Alburquerque R., Kato Y.P., Arrieta E., Peralta A.C., Palmberg P., Parrish R.K. (2016). Three-year follow-up of a novel aqueous humor MicroShunt. J. Glaucoma.

[10] Arora K.S., Robin A.L., Corcoran K.J., Corcoran S.L., Ramulu P.Y. (2015). Use of various glaucoma surgeries and procedures in Medicare beneficiaries from 1994 to 2012. Ophthalmology.

[11] Desai M.A., Gedde S.J., Feuer W.J., Shi W., Chen P.P., Parrish R.K. (2011). Practice preferences for glaucoma surgery: A survey of the American Glaucoma Society in 2008. Ophthalmic Surg. Lasers Imaging.

[12] Vinod K., Gedde S.J., Feuer W.J., Panarelli J.F., Chang T.C., Chen P.P., Parrish R.K. (2017). Practice preferences for glaucoma surgery: A survey of the American Glaucoma Society. J. Glaucoma.

[13] Rodriguez-Una I., Azuara-Blanco A., King A.J. (2017). Survey of glaucoma surgical preferences and post-operative care in the United Kingdom. Clin. Exp. Ophthalmol..

[14] Fujita A., Hashimoto Y., Matsui H., Yasunaga H., Aihara M. (2022). Recent trends in glaucoma surgery: A nationwide database study in Japan, 2011–2019. Jpn. J. Ophthalmol..

[15] Tanito M. (2023). Nationwide analysis of glaucoma surgeries in fiscal years of 2014 and 2020 in Japan. J. Pers. Med..

[16] Kono Y., Kasahara M., Hirasawa K., Tsujisawa T., Kanayama S., Matsumura K., Morita T., Shoji N. (2020). Long-term clinical results of trabectome surgery in patients with open-angle glaucoma. Graefes Arch. Clin. Exp. Ophthalmol..

[17] Richter G.M., Takusagawa H.L., Sit A.J., Rosdahl J.A., Chopra V., Ou Y., Kim S.J., WuDunn D. (2024). Trabecular Procedures Combined with Cataract Surgery for Open-Angle Glaucoma: A Report by the American Academy of Ophthalmology. Ophthalmology.

[18] Tanito M., Sugihara K., Tsutsui A., Hara K., Manabe K., Matsuoka Y. (2021). Midterm Results of Microhook ab Interno trabeculotomy in Initial 560 Eyes with Glaucoma. J. Clin. Med..

[19] Iwasaki K., Arimura S., Takihara Y., Takamura Y., Inatani M. (2018). Prospective cohort study of corneal endothelial cell loss after Baerveldt glaucoma implantation. PLoS ONE..

[20] Ibarz-Barberá M., Morales-Fernández L., Corroto-Cuadrado A., Martinez-Galdón F., Tañá-Rivero P., Gómez de Liaño R., Teus M.A. (2022). Corneal Endothelial Cell Loss After PRESERFLO™ MicroShunt Implantation in the Anterior Chamber: Anterior Segment OCT Tube Location as a Risk Factor. Ophthalmol. Ther..

[21] Panarelli J.F., Moster M.R., Garcia-Feijoo J., Flowers B.E., Baker N.D., Barnebey H.S., Grover D.S., Khatana A.K., Lee B., Nguyen T. (2024). Ab-Externo MicroShunt versus trabeculectomy in Primary Open-Angle Glaucoma: Two-year Results from a Randomized, Multicenter Study. Ophthalmology.

[22] Iwase A., Suzuki Y., Araie M., Yamamoto T., Abe H., Shirato S., Kuwayama Y., Mishima H.K., Shimizu H., Tomita G. (2004). The prevalence of primary open-angle glaucoma in Japanese: The Tajimi Study. Ophthalmology.

[23] Oie S., Ishida K., Yamamoto T. (2017). Impact of intraocular pressure reduction on visual field progression in normal-tension glaucoma followed up over 15 years. Jpn. J. Ophthalmol..

[24] Nakajima K., Sakata R., Ueda K., Fujita A., Fujishiro T., Honjo M., Shirato S., Aihara M. (2021). Central visual field change after fornix-based trabeculectomy in Japanese normal-tension glaucoma patients managed under 15 mmHg. Graefes Arch. Clin. Exp. Ophthalmol..

[25] Christakis P.G., Zhang D., Budenz D.L., Barton K., Tsai J.C., Ahmed I.I.K., ABC-AVB Study Groups (2017). Five-year pooled data analysis of the Ahmed Baerveldt comparison study and the Ahmed versus Baerveldt study. Am. J. Ophthalmol..

[26] Iwasaki K., Kojima S., Wajima R., Matsuda A., Yoshida K., Tsutsui A., Kono M., Nozaki M., Namiguchi K., Nitta K. (2025). Surgical Outcomes of Baerveldt Glaucoma Implant Versus Ahmed Glaucoma Valve in Neovascular Glaucoma: A Retrospective Multicenter Study. Adv. Ther..

[27] Marquardt D., Lieb W.E., Grehn F. (2004). Intensified postoperative care versus conventional follow-up: A retrospective long-term analysis of 177 trabeculectomies. Graefes Arch. Clin. Exp. Ophthalmol..

[28] Panarelli J.F., Nayak N.V., Sidoti P.A. (2016). Postoperative management of trabeculectomy and glaucoma drainage implant surgery. Curr. Opin. Ophthalmol..

[29] Chang T.C., Vanner E.A., Parrish R.K. (2019). Glaucoma surgery preferences when the surgeon adopts the role of the patient. Eye.

[30] Amaral D.C., Louzada R.N., Moreira P.H.S., de Oliveira L.N., Yuati T.T., Guedes J., Alves M.R., Mora-Paez D.J., Monteiro M.L.R. (2024). Combined endoscopic cyclophotocoagulation and phacoemulsification versus phacoemulsification alone in the glaucoma treatment: A systematic review and meta-analysis. Cureus.

[31] de Crom R.M.P.C., Kujovic-Aleksov S., Webers C.A.B., Berendschot T.T.J.M., Beckers H.J.M. (2025). Long-term treatment outcomes of micropulse transscleral cyclophotocoagulation in primary and secondary glaucoma: A 5-year analysis. Ophthalmol. Ther..

