# Supplementary material for: Clinical Practice Preferences for Glaucoma Surgery in Japan in 2024
Source: J Clin Med. 2025 Mar 17;14(6):2039. doi: 10.3390/jcm14062039 (PMC11942949; doi:10.3390/jcm14062039)
Supplement: Supplementary file 1 [file jcm-14-02039-s001.zip › jcm-3487178-supplementary.pdf]

Supplemental Table S1. Which glaucoma surgeries had you performed for the following clinical scenarios in 2024?

Clinical scenario	1	Unoperated mild to moderate POAG (MD > −12 dB) eyes associated with cataract
	2	Unoperated advanced POAG (MD ≤ −12 dB) eyes associated with cataract
	3	Unoperated mild to moderate POAG (MD > −12 dB) eyes without cataract
	4	Unoperated advanced POAG (MD ≤ −12 dB) eyes without cataract
	5	Pseudophakic mild to moderate POAG (MD > −12 dB) eyes with prior corneal incision phacoemulsification
	6	Pseudophakic advanced POAG (MD ≤ −12 dB) eyes with prior corneal incision phacoemulsification
	7	Pseudophakic advanced POAG (MD ≤ −12 dB) eyes with a previous failed trabeculectomy
	8	Pseudophakic advanced POAG (MD ≤ −12 dB) eyes with two failed trabeculectomies
	9	Unoperated mild to moderate NTG (MD > −12 dB) eyes associated with cataract
	10	Unoperated advanced NTG (MD ≤ −12 dB) eyes associated with cataract
	11	Unoperated advanced NTG (MD ≤ −12 dB) eyes without cataract
	12	Pseudophakic advanced NTG (MD ≤ −12 dB) eyes with prior corneal incision phacoemulsification
	13	Pseudophakic advanced NTG (MD ≤ −12 dB) eyes with a previous failed trabeculectomy
	14	Pseudophakic advanced NTG (MD ≤ −12 dB) eyes with two failed trabeculectomies
	15	Unoperated UG eyes associated with cataract
	16	Unoperated UG eyes without cataract
	17	Pseudophakic UG eyes
	18	Pseudophakic UG eyes with a previous failed trabeculectomy
	19	Pseudophakic NVG eyes with prior vitrectomy and panretinal photocoagulation
	20	Pseudophakic NVG eyes with prior vitrectomy, panretinal photocoagulation, and a previous failed trabeculectomy

MD, mean deviation; NTG, normal tension glaucoma; NVG, neovascular glaucoma; POAG, primary open-angle glaucoma; UG, uveitic glaucoma.

Supplemental Table S2. Surgical preferences for each type of glaucoma across all clinical settings.

Type of glaucoma surgery	POAG								NTG						UG			NVG		
	Unoperated eyes with cataract		Unoperated eyes without cataract		Pseudophakic eyes with a corneal incision phaco		Pseudophakic eyes with a previous failed trabeculectomy	Pseudophakic eyes with two failed trabeculectomies	Unoperated eyes with cataract		Unoperated eyes without cataract	Pseudophakic eyes with a corneal incision phaco	Pseudophakic eyes with a previous failed trabeculectomy	Pseudophakic eyes with two failed trabeculectomies	Unoperated eyes with cataract	Unoperated eyes without cataract	Pseudophakic eyes	Pseudophakic eyes with a previous failed trabeculectomy	Pseudophakic eyes with prior vitrectomy and panretinal photocoagulation	Pseudophakic eyes with prior vitrectomy, panretinal photocoagulation, and a previous failed trabeculectomy
	Mild to moderate (%)	Advanced (%)	Mild to moderate (%)	Advanced (%)	Mild to moderate (%)	Advanced (%)	Advanced (%)	Advanced (%)	Mild to moderate (%)	Advanced (%)	Advanced (%)	Advanced (%)	Advanced (%)	Advanced (%)						
Microhook+Phaco	51.8	24.8	2.4	2.4	0.0	0.0	0.0	0.0	37.5	21.6	1.2	0.0	0.0	0.0	20.2	1.2	0.0	0.0	0.0	0.0
Microhook	0.0	0.0	50.8	5.9	48.4	4.7	0.8	0.8	0.0	0.0	4.5	0.8	0.8	0.8	2.4	14.3	13.9	3.2	0.0	0.0
iStent inject W+Phaco	18.1	3.3	1.2	1.2	0.0	0.0	0.0	0.0	14.5	4.1	1.2	0.0	0.0	0.0	0.0	0.0	0.0	0.0	0.0	0.0
iStent inject W	0.0	0.0	0.8	0.8	0.8	0.8	0.0	0.0	0.0	0.0	0.8	0.8	0.0	0.0	0.0	0.0	0.8	0.0	0.0	0.0
KDB+Phaco	7.1	3.6	0.0	0.0	0.0	0.0	0.0	0.0	4.8	2.4	0.0	0.0	0.0	0.0	4.8	0.0	0.0	0.0	0.0	0.0
KDB	0.0	0.0	9.5	0.0	7.2	0.0	0.0	0.0	0.0	0.0	0.0	0.0	0.0	0.0	0.0	2.4	2.4	0.0	0.0	0.0
Suture trabeculotomy+Phaco	6.7	1.4	0.0	0.0	0.0	0.0	0.0	0.0	2.0	0.6	0.0	0.0	0.0	0.0	5.1	0.0	0.0	0.0	0.0	0.0
Suture trabeculotomy	0.0	0.0	6.7	1.6	7.9	1.6	0.8	0.6	0.0	0.0	0.8	0.8	0.8	0.8	0.0	4.3	5.1	0.6	0.0	0.0
HMS+Phaco	6.1	2.4	0.0	0.0	0.0	0.0	0.0	0.0	6.1	2.4	0.0	0.0	0.0	0.0	1.2	0.0	0.0	0.0	0.0	0.0
Other MIGS+Phaco	4.8	1.2	0.0	0.0	0.0	0.0	0.0	0.0	2.4	0.0	0.0	0.0	0.0	0.0	2.4	1.2	0.0	0.0	0.0	0.0
Other MIGS	0.0	0.0	6.0	0.8	4.8	0.8	0.0	0.0	0.0	0.0	0.0	0.0	0.0	0.0	0.0	2.4	2.4	0.0	0.0	0.0
Phaco only	0.0	0.0	0.0	0.0	0.0	0.0	0.0	0.0	2.4	0.0	0.0	0.0	0.0	0.0	0.0	0.0	0.0	0.0	0.0	0.0
PMS+Phaco	4.8	28.8	0.0	0.0	0.0	0.0	0.0	0.0	20.2	17.3	0.0	0.0	0.0	0.0	7.2	0.0	0.0	0.0	0.0	0.0
PMS	0.0	1.6	17.5	32.9	23.4	40.1	18.2	7.1	2.4	0.8	19.1	25.8	11.1	9.9	2.4	10.7	11.5	13.1	8.4	4.8
Trabeculectomy +Phaco	0.0	20.8	0.0	0.0	0.0	0.0	0.0	0.0	7.1	36.3	0.0	0.0	0.0	0.0	32.5	0.0	0.0	0.0	0.0	0.0
Trabeculectomy	0.6	9.7	5.1	52.0	7.5	49.6	57.2	15.7	0.6	12.1	72.4	69.4	75.0	18.7	20.2	62.7	57.2	53.7	47.2	19.8
ExPRESS+Phaco	0.0	2.4	0.0	0.0	0.0	0.0	0.0	0.0	0.0	2.4	0.0	0.0	0.0	0.0	0.0	0.0	0.0	0.0	0.0	0.0
ExPRESS	0.0	0.0	0.0	2.4	0.0	2.4	2.4	0.0	0.0	0.0	0.0	2.4	2.4	2.4	0.0	0.0	0.0	0.0	1.2	0.0
AGV+Phaco	0.0	0.0	0.0	0.0	0.0	0.0	0.0	0.0	0.0	0.0	0.0	0.0	0.0	0.0	1.6	0.0	0.0	0.0	0.0	0.0
AGV	0.0	0.0	0.0	0.0	0.0	0.0	10.7	49.0	0.0	0.0	0.0	0.0	2.4	34.1	0.0	0.8	7.5	20.8	34.1	62.7
BGI	0.0	0.0	0.0	0.0	0.0	0.0	4.8	20.4	0.0	0.0	0.0	0.0	2.4	20.6	0.0	0.0	0.0	4.8	9.1	10.3
Bleb revision or needling	0.0	0.0	0.0	0.0	0.0	0.0	5.1	1.6	0.0	0.0	0.0	0.0	5.1	9.5	0.0	0.0	0.0	3.8	0.0	2.4
MP-TSCPC	0.0	0.0	0.0	0.0	0.0	0.0	0.0	4.8	0.0	0.0	0.0	0.0	0.0	3.2	0.0	0.0	0.0	0.0	0.0	0.0

AGV, Ahmed glaucoma valve; BGI, Baerveldt glaucoma implant; HMS, Hydrus Microstent; KDB, Kahook dual blade; MIGS, minimally invasive glaucoma surgery; MP-TSCPC, micropulse transscleral cyclophotocoagulation; NTG, normal tension glaucoma; NVG, neovascular glaucoma; Phaco, phacoemulsification; PMS, PreserFlo MicroShunt; POAG, primary open-angle glaucoma; UG, uveitic glaucoma.
